# Visual Evaluation of Ultrafast MRI in the Assessment of Residual Breast Cancer After Neoadjuvant Systemic Therapy: A Preliminary Study Association with Subtype

**DOI:** 10.3390/tomography8030125

**Published:** 2022-06-10

**Authors:** Maya Honda, Masako Kataoka, Mami Iima, Rie Ota, Akane Ohashi, Ayami Ohno Kishimoto, Kanae Kawai Miyake, Marcel Dominik Nickel, Yosuke Yamada, Masakazu Toi, Yuji Nakamoto

**Affiliations:** 1 Department of Diagnostic Imaging and Nuclear Medicine, Kyoto University Graduate School of Medicine, 54 Shogoin-Kawaharacho, Kyoto 606-8507, Japan; mayah.217@gmail.com (M.H.); mamiiima@kuhp.kyoto-u.ac.jp (M.I.); ota_rie0624@kuhp.kyoto-u.ac.jp (R.O.); kanaek@kuhp.kyoto-u.ac.jp (K.K.M.); ynakamo1@kuhp.kyoto-u.ac.jp (Y.N.); 2 Department of Diagnostic Radiology, Kansai Electric Power Hospital, 2-1-7, Fukushima, Osaka 553-0003, Japan; 3 Institute for Advancement of Clinical and Translational Science (iACT), Kyoto University Hospital, 4 Shogoin-Kawaharacho, Kyoto 606-8507, Japan; 4 Department of Translational Medicine, Diagnostic Radiology, Lund University, Skåne University Hospital, 205-02 Malmo, Sweden; amaoh135@gmail.com; 5 Department of Diagnostic Radiology, Kyoto Katsura Hospital, Yamadahirao-cho, Kyoto 615-8256, Japan; akohno1980@gmail.com; 6 MR Application Predevelopment, Siemens Healthcare GmbH, Allee am Roethelheimpark 2, 91052 Erlangen, Germany; marcel.nickel@siemens-healthineers.com; 7 Department of Diagnostic Pathology, Kyoto University Hospital, 54 Shogoin-Kawaharacho, Kyoto 606-8507, Japan; yyamada@kuhp.kyoto-u.ac.jp; 8 Department of Breast Surgery, Kyoto University Graduate School of Medicine, 54 Shogoin-Kawaharacho, Kyoto 606-8507, Japan; toi@kuhp.kyoto-u.ac.jp

**Keywords:** breast neoplasm, magnetic resonance imaging, treatment

## Abstract

The purpose of this study was to investigate the diagnostic performance of ultrafast dynamic contrast-enhanced (UF-DCE) MRI after the completion of neoadjuvant systemic therapy (NST) in breast cancer. In this study, MR examinations of 55 post-NST breast cancers were retrospectively analyzed. Residual tumor sizes were measured in the 20th phase of UF-DCE MRI, early and delayed phases of conventional dynamic contrast-enhanced (DCE) MRI, and high spatial-resolution contrast enhanced MRI (UF, early, delayed, and HR, respectively). The diagnostic performance for the detection of residual invasive cancer was calculated by receiver operating characteristic (ROC) analysis. The size difference between MRI and pathological findings was analyzed using the Wilcoxon signed-rank test with the Bonferroni correction. The overall area under the ROC curve (AUC) was highest for UF (0.86 and 0.88 for readers 1 and 2, respectively). The difference in imaging and pathological sizes for UF (5.7 ± 8.2 mm) was significantly smaller than those for early, delayed, and HR (*p* < 0.01). For luminal subtype breast cancer, the size difference was significantly smaller for UF and early than for delayed (*p* < 0.01). UF-DCE MRI demonstrated higher AUC and specificity for the more accurate detection of residual cancer and the visualization of tumor extent than conventional DCE MRI.

## 1. Introduction

Neoadjuvant systemic therapy (NST) is a well-established treatment for breast cancer that can downstage the tumor for less-invasive surgery and provide information on response to chemotherapy [1]. Pathological complete response (pCR) after NST is an important prognostic factor, especially in triple-negative breast cancer (TNBC) and human epidermal growth factor receptor 2 (HER2)-enriched breast cancer (HER2+BC) [2].

In previous studies, surgery was considered unnecessary in patients whose cancer had disappeared after NST [3,4]. However, early attempts to avoid surgery resulted in increased local recurrence because the determination of clinical complete response (cCR) based on palpation and conventional imaging modalities [3,5] was not as accurate as the determination of cCR based on MRI [6]. Recently, a trial for non-surgical treatment started enrolling Stage I or II HER2+BC or TNBC patients, in whom complete response after NST was determined by image-guided biopsy [7].

Because pCR can only be confirmed after surgery, cCR is used as an alternative for preoperative treatment planning. Although a biopsy is often useful, it sometimes fails to sample the viable portion of a heterogeneous tumor, whereas dynamic contrast-enhanced (DCE) MRI can evaluate the entire residual lesion. Meanwhile, the performance of MRI for the prediction of pCR has an unsatisfactory accuracy of around 74% [8]. Furthermore, enhancing scarring or inflammatory tissue is sometimes misdiagnosed as residual cancer [9]. The improved accuracy of MRI in predicting pCR may lead to the establishment of non-surgical treatment options for breast cancer patients.

**Figure 1:**
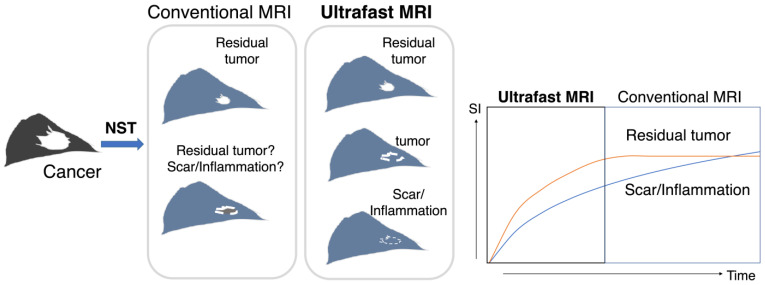
A schema of the study design/hypothesis. This schema shows the treatment course of NST, imaging evaluation, surgery, and the final comparison of post-NST image with surgical specimen. Both residual tumor and scarring/inflammation were enhanced on conventional dynamic contrast-enhanced (DCE) MRI, while ultrafast DCE MRI may differentiate the hypervascular viable residual tumor from slow-enhancing scarring/inflammation. NST: neoadjuvant systemic treatment; SI: signal intensity.

Ultrafast DCE (UF-DCE) MRI is an emerging protocol with high temporal and spatial resolution. It can capture the inflow of breast lesions, which is useful for distinguishing cancer tissue with fast initial enhancement from benign or normal breast tissue with slow enhancement. UF-DCE MRI provided improved conspicuity of cancer in the very early phase, before the enhancement of benign or normal parenchyma was observed [10,11,12,13,14,15]. In the post NAC status, the residual tumor is enhanced weaker than before, which overlaps the slow enhancement of post-treatment inflammation or scarring and causes overestimation of residual tumor. We hypothesized that UF-DCE MRI may perform better in distinguishing rapidly enhancing viable cancer tissue from gradually enhancing scarring or inflammatory tissue after NST compared with conventional DCE MRI (Figure 1).

Recently, some studies investigated the use of UF-DCE MRI to assess the breast cancer response to NST. Kim et al., found an association between UF-DCE MRI-derived parameters and pCR among triple-negative breast cancer patients [16]. According to one study, evaluating the diagnostic performance of UF-DCE MRI to detect residual cancer after NST, UF-DCE MRI was more challenging than evaluating conventional DCE MRI [17]. Image findings after NST may differ among breast cancer subtypes, which may affect the diagnostic performance of UF-DCE MRI. Therefore, the present study aimed to determine the value of UF-DCE MRI for evaluating the post-NST status of breast cancer in association with the subtype.

## 2. Materials and Methods

### 2.1. Patients and MRI Setting

This retrospective study was approved by our institutional review board, with a waiver of the requirement for informed consent.

**Figure 2:**
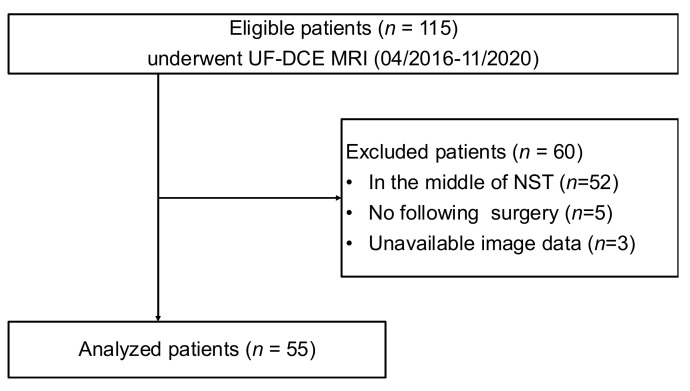
The flow diagram of the case collection and selection procedure. UF-DCE MRI: ultrafast dynamic contrast-enhanced MRI; NST: neoadjuvant systemic treatment.

A search of our MRI database identified 115 consecutive breast MR scans for NST evaluation using the UF-DCE protocol from April 2016 to October 2020. From these, 60 scans were excluded for the following reasons: in the middle of NST (*n* = 52), no surgery (*n* = 5), and unavailable image data (*n* = 3). Consequently, 55 female patients (mean age, 49.7 years; range, 26–77 years) who underwent NST for invasive breast cancer and pre-surgical DCE MRI with the UF-DCE protocol were enrolled (Figure 2).

**Figure 3:**
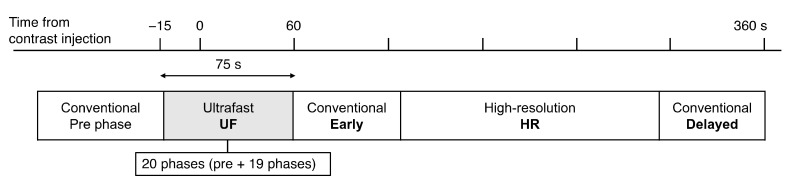
The timing of each acquisition.

All MRI examinations were performed using a 3T scanner (MAGNETOM Prisma and Skyra; Siemens Healthineer, Erlangen, Germany) with 16- or 18-channel dedicated breast coil. Gadobutrol (Gadovist; Bayer, Berlin, Germany) was intravenously infused at a dose of 0.1 mL/kg and a rate of 2.0 mL/s, followed by 20 mL of saline at the same rate. The DCE MRI protocols were as follows: pre-contrast; UF-DCE MRI, 15 s before to 60 s after contrast injection; 2 s preparation time followed by 3.7 s/phase × 20 phases; early phase of DCE MRI, 60–120 s after contrast injection (early); high spatial-resolution contrast-enhanced MRI, 120–300 s after contrast injection (HR); and delayed phase of DCE MRI, 300–360 s after contrast injection (delayed) (Figure 3). UF-DCE MRI was performed using a prototype based on a 3D gradient-echo volumetric interpolated breath-hold examination sequence with a compressed sensing reconstruction (acceleration, 16.5; iteration number, 30). The images for the 20th phase of UF-DCE MRI (UF), early, delayed, and HR were evaluated. Table 1 summarizes the sequence parameters.

### 2.2. Image Evaluation

Two independent radiologists, reader 1 (M.H., a board-certified breast radiologist for 10 years) and reader 2 (M.K., a board-certified breast radiologist for 20 years), evaluated the residual enhancing area on the UF, early, delayed, and HR images, in that order. The readers were allowed to refer to the pre-NST MR images. The tumor size and morphology on the pre-NST MR images and patterns of shrinkage were evaluated. The tumor size was defined as the maximum length of the tumor on the axial plane. When no enhancing area was observed, the tumor was considered CR (size = 0). The pattern of shrinkage was classified as “concentric” or “non-concentric”. Concentric shrinkage included simple concentric shrinkage and concentric shrinkage to small foci of <5 mm after NST [18]. The two readers reviewed discordant cases together until they reached a consensus.

### 2.3. Histopathological Assessment

Tumor subtypes were obtained from histopathology reports on core needle biopsies performed before NST. Estrogen receptor (ER) expression was defined as ≥1% positive cells on immunohistochemical (IHC) examination or Allred score ≥ 3. HER2 positivity was defined as 3+ score on IHC examination or determined by in situ hybridization in cases with 2+ score on IHC examination. ER-positive cancers were classified as luminal subtype; ER-negative and HER2-positive cancers were classified as HER2+BC; and cancers with neither ER nor HER2 positivity were classified as TNBC. Pathological responses were obtained from histopathology reports on surgical specimens, with pCR defined as non-invasive cancer, allowing in situ carcinoma.

### 2.4. Statistical Analysis

Statistical analyses were performed with R (version 4.0.3; The R Foundation for Statistical Computing, Vienna, Austria) or MedCalc (version 15.1.4; MedCalc, Mariakerke, Belgium). The Wilcoxon rank-sum test and Fisher’s exact test were used to compare variables (patient age, tumor subtype, morphology, size, and shrinkage pattern) between the pCR and non-pCR groups. A value of *p* < 0.05 was considered statistically significant. The inter-reader agreement for the presence of a residual lesion on each protocol was evaluated by kappa statistics. The kappa values were assessed as follows [19]: ≤0.2, slight agreement; 0.21–0.40, fair agreement; 0.41–0.60, moderate agreement; 0.61–0.80, substantial agreement; and 0.81–0.99, almost perfect agreement. Receiver operating characteristic (ROC) analysis was performed to determine the area under the ROC curve (AUC) of each protocol for the presence of a residual lesion. The DeLong method with the Bonferroni correction was performed for pairwise comparisons of ROC curves using easy ROC, a web tool for ROC analysis [20]. The significance level after the Bonferroni correction was defined as *p* < 0.05/3 = 0.0167. The inter-reader agreement for the lesion diameter was evaluated by intraclass correlation coefficient (ICC) calculation. The ICCs were assessed as follows [21]: <0.50, poor agreement; 0.50–0.75, moderate agreement; 0.75–0.90, good agreement; and >0.90, excellent agreement. The size of the residual lesion on each protocol was compared with that in the surgical specimen, and the difference between the imaging and pathological sizes on each protocol was compared using the Wilcoxon signed-rank test with the Bonferroni correction. The absolute value of “imaging size minus pathological size” was determined to be the “difference”. If the pathological lesion size was <1 mm, it was calculated as 1 mm. The significance level after the Bonferroni correction was defined as *p* < 0.05/3 = 0.0167. The average values of the two readers were used for the analysis.

## 3. Results

A total of 55 lesions in 55 patients were evaluated, of which 22 lesions achieved pCR. The patient characteristics are summarized in Table 2. Among the patients, 33 received anthracycline and taxane-based chemotherapy, 15 received anti-HER2 therapy with chemotherapy, and 3 received endocrine therapy with cyclin-dependent kinases 4 and 6 (CDK4/6) inhibitor. The remaining four patients were treated with other regimens. The mean interval between MRI and surgery was 14.5 days (range, 1–48 days).

**Figure 4:**
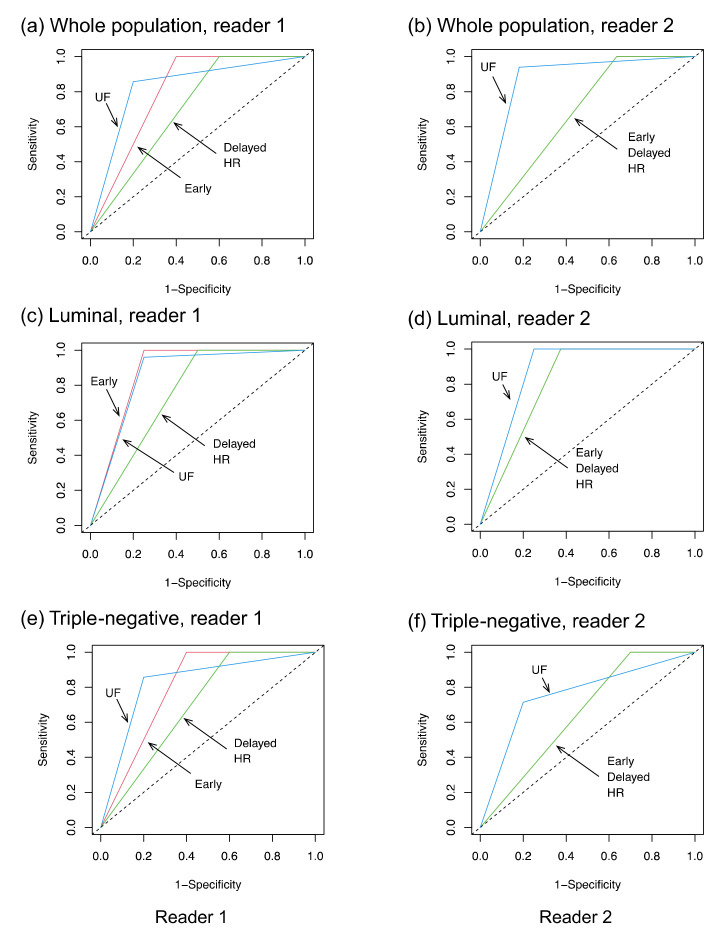
The receiver operating characteristic curves. UF: the 20th phase of ultrafast dynamic contrast-enhanced MRI; early: the early phase; HR: high spatial-resolution; and delayed: delayed phase.

The inter-reader agreement for the presence or absence of a residual lesion was better for UF (kappa = 0.81) than for early, delayed, and HR (kappa = 0.64, 0.68, and 0.52, respectively). Table 3 summarizes the diagnostic performances. The overall AUC on UF (0.86 and 0.88 for reader 1 and reader 2, respectively) was higher than those on early, delayed, and HR (0.80, 0.70, and 0.70 for reader 1, and 0.68, 0.68, and 0.68 for reader 2, respectively) (Figure 4). There was a significant difference between UF and early, delayed, or HR as rated by reader 2. A sub-analysis by subtype revealed no significant difference in terms of AUC, but the specificity on UF (0.80 for both readers) tended to be higher than those on early, delayed, and HR (0.60, 0.40, and 0.40 for reader 1, and 0.30, 0.30, and 0.30 for reader 2, respectively) among TNBC cases. The false-negative cases on UF were one luminal subtype (reader 1, 1 mm of invasive cancer), one HER2+BC (reader 1, 6 mm of invasive cancer), and two TNBC (reader 2, 20 mm of invasive cancer; readers 1 and 2, 5 mm of invasive cancer). All false-negative cases were masses on pre-NST MRI, and all but one of these cases showed a concentric shrinkage pattern.

**Figure 5:**
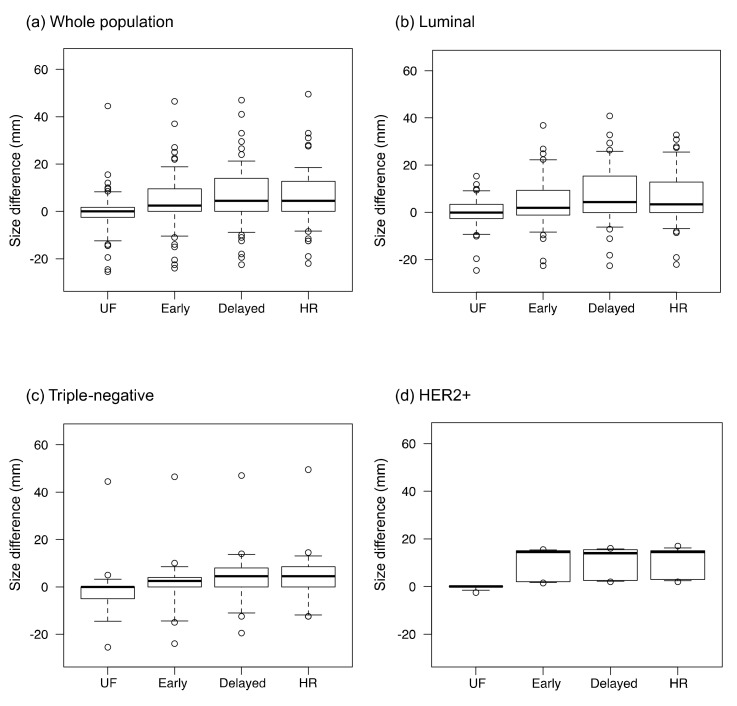
The size differences between the imaging and pathological findings. The values were calculated as the residual lesion size on each protocol minus the pathological lesion size. UF: the 20th phase of ultrafast dynamic contrast-enhanced MRI; early: the early phase; HR: the high spatial-resolution; delayed: the delayed phase; and HER2+: the estrogen receptor-negative and human epidermal growth factor receptor-2-positive. The dots on the boxplots are outliers.

**Figure 6:**
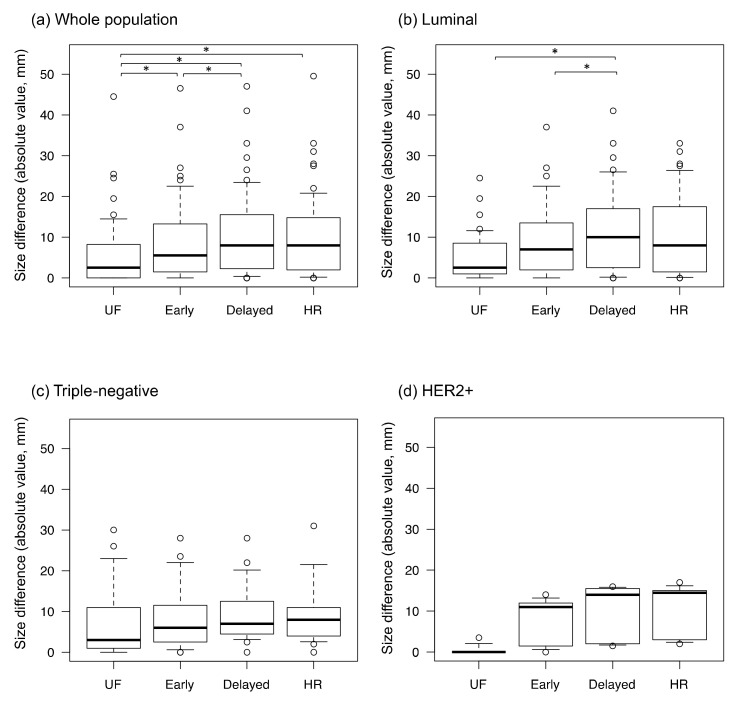
The absolute values of the size differences between the imaging and pathological findings. UF: the 20th phase of ultrafast dynamic contrast-enhanced MRI; early: the early phase; HR: high spatial-resolution; delayed: the delayed phase; and HER2+: estrogen receptor-negative and human epidermal growth factor receptor-2-positive. * represents a statistically significant difference. The dots on the boxplots are outliers.

The inter-reader agreement for lesion diameter was excellent on all protocols (ICC: 0.92–0.98). The differences for imaging size compared with pathological size, calculated as the imaging size on each protocol minus the pathological size, are shown in Figure 5. The absolute size differences between the imaging and pathological findings are shown in Figure 6. The difference between the imaging and pathological sizes on UF (5.7 ± 8.2 mm) was significantly smaller than those on early, delayed, and HR (8.9 ± 9.9 mm, 10.6 ± 10.4 mm, and 10.1 ± 26.7 mm, respectively, *p* < 0.01). Among the luminal subtype cancers, the size difference was significantly smaller on UF and early (5.5 ± 5.9 mm and 9.4 ± 9.5 mm, respectively) than on delayed (11.4 ± 10.6 mm, *p* < 0.01). 

**Figure 7:**
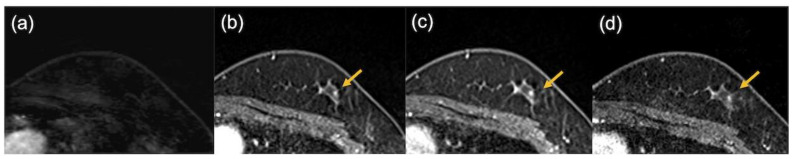
The images of a patient with triple-negative breast cancer after neoadjuvant chemotherapy. Pathological evaluation was complete response. No enhancing area was detected on the ultrafast dynamic contrast-enhanced MR image (**a**), while enhancing foci were observed in the early phase (**b**), the delayed phase (**c**), and the high spatial-resolution (**d**) images (arrows).

**Figure 8:**
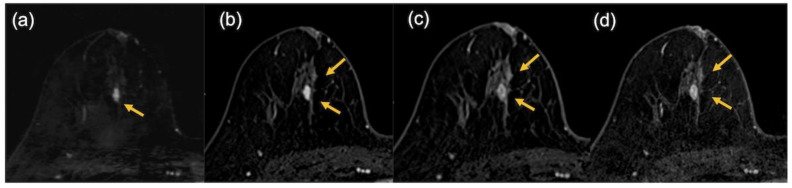
The images of a patient with luminal subtype breast cancer after neoadjuvant endocrine therapy in combination with cyclin-dependent kinases 4 and 6 inhibitor. In the pathological findings, 12 mm of invasive cancer remained. In the MRI findings, the lesion was evaluated as being slightly smaller on the ultrafast dynamic contrast-enhanced MR image (9.5 mm) (**a**) but larger in the early phase (21.5 mm) (**b**), the delayed phase (24 mm) (**c**), and the high spatial-resolution (24.5 mm) (**d**) images (arrows).

Representative cases are shown in Figure 7 and Figure 8.

## 4. Discussion

The present results show the potential of UF-DCE MRI for evaluating the post-NST status of breast cancer. With the use of UF-DCE MRI, higher AUC and specificity can be obtained with equivalent sensitivity for the detection of residual cancer than conventional DCE MRI. The final phase of UF-DCE MRI may be an appropriate time to capture the enhancement of residual invasive cancer. The short scanning time is another benefit of UF-DCE MRI for post-NST evaluation.

The present findings differ from those in a previous report that argued the need to observe late enhancement for pCR prediction [22]. The discrepancy may be due to the different definitions of pCR, with in situ lesions allowed or disallowed. A previous study found no significant difference between a residual ductal carcinoma in situ (DCIS) population and a non-residual invasive or in situ disease population in terms of 61-month disease-free survival and 5-year overall survival [23]. A systematic review further found no significant difference in local recurrence rates for DCIS patients who underwent surgery and postoperative radiotherapy between those with clear margins and those with involved margins [24]. These results suggest that residual DCIS can be acceptable after surgery. Kim and colleagues compared the diagnostic performances of the conventional early and delayed phases for the determination of pCR and found a higher AUC in the early phase [25]. Our findings are noteworthy in that UF-DCE MRI can be useful for the detection of invasive disease.

Another study compared ultrafast and standard DCE MRI for the evaluation of breast cancer after NST [17] and found that UF-DCE MRI had higher specificity, which is consistent with the present results. Improved specificity should be an advantage of UF-DCE MRI. Meanwhile, UF-DCE MRI was found to have a lower AUC in the same study. This difference from the present results may partly arise from differences in the treatment regimens, UF-DCE MRI protocols, and contrast agents. A recent study evaluated the functional tumor volume (FTV) in TNBC patients and revealed that FTV at 1 min after injection provided better discrimination between pCR and non-pCR than FTV at 2.5 min after injection [26]. We further demonstrated that UF-DCE MRI can visualize invasive lesions more accurately than conventional DCE MRI, consistent with a previous study [17]. The evaluation of the extent of residual cancer is important for determining the appropriate surgical approach [27]. DCE MRI is the most accurate imaging modality for evaluation of tumor size after NST [28], but the optimal timing of the scan remains to be determined. UF-DCE MRI may be a promising candidate for the measurement of residual invasive cancer.

The sub-analysis by subtype revealed that for the luminal subtype, UF-DCE MRI can significantly reduce the size error for imaging findings relative to pathological findings compared with conventional DCE MRI. A previous study found that the difference in lesion size between DCE MRI and pathological findings was greater for the luminal subtype than for TNBC or HER+BC [29]. Therefore, the use of UF-DCE MRI may improve the size evaluation for luminal subtype cancers after NST.

The de-escalation of surgery is discussed for patients showing CR after NST. In a study on 121 post-NST patients, patients with radiological CR (*n* = 28) did not undergo surgery but did receive radiotherapy. The patients in this “no surgery” group showed better overall survival and disease-free survival than patients who did not achieve radiological CR and underwent wide-local excision or mastectomy [30]. Because breast surgery affects women’s mental health [31], breast preservation with minimal surgical intervention is preferable. Although MRI-based CR requires confirmation by a biopsy [32], the accurate prediction of pCR using UF-DCE MRI may be helpful in selecting cohorts for de-escalated surgical management. 

The limitations of the present study include the retrospective single-site study design and the small number of patients. Future studies including a sufficient number of cases are needed to clarify the use of UF-DCE MRI in the evaluation of treatment response in each subtype, especially TNBC and HER2+BC. Quantitative variables such as maximum slope or time to enhancement were not included. However, qualitative assessments involving the placement of a region of interest are often difficult on shrunken cancer lesions after NST.

In conclusion, UF-DCE MRI demonstrated higher AUC for the more accurate detection of residual cancer and the visualization of tumor extent compared with conventional DCE MRI. UF-DCE MRI may be a promising method for evaluating the post-NST status of breast cancer.

## Figures and Tables

**Table 1:** The MRI sequence parameters.

	UF	Early, Delayed	HR
Orientation	Axial	Axial	Coronal
Sequence	VIBE without FS	VIBE with FS	VIBE with FS
TR, ms	4.80	3.84	4.61
TE, ms	2.46	1.43	1.80
FOV, mm^2^	360 × 360	330 × 330	330 × 330
Matrix	384 × 269	384 × 346	512 × 461
Thickness, mm	2.5	1	0.8
Slices	60	144	176
Temporal resolution, s	3.65	60	146

UF, ultrafast dynamic contrast-enhanced MRI; HR, high-resolution contrast-enhanced MRI; TR, repetition time; TE, echo time; FOV, field of view; FS, fat suppression; and VIBE, volumetric interpolated breath-hold examination.

**Table 2:** The patient characteristics.

Variables	*n*	Overall, *n* = 55 ^1^	pCR, *n* = 22 ^1^	Non-pCR, *n* = 33 ^1^	*p*-Value ^2^
Age (years)	55	49.7 ± 11.7	48.6 ± 9.7	50.5 ± 12.9	0.8
Subtype	55				0.007
Luminal		33 (60%)	8 (36%)	25 (76%)	
HER2+		5 (9.1%)	4 (18%)	1 (3.0%)	
TN		17 (31%)	10 (45%)	7 (21%)	
pre-NST size (mm)	54 ^3^	40.5 ± 23.7	28.9 ± 19.8	48.5 ± 23.0	<0.001
Morphology	54 ^3^				0.5
mass		43 (80%)	19 (86%)	24 (75%)	
NME		11 (20%)	3 (14%)	8 (25%)	
Shrink pattern	54 ^3^				0.004
CR		5 (9.3%)	5 (23%)	0 (0%)	
CS		24 (44%)	12 (55%)	12 (38%)	
non-CS		17 (31%)	4 (18%)	13 (41%)	
SD		8 (15%)	1 (4.5%)	7 (22%)	

Data in columns 2 and 3 are the number of patients with the percentage in parentheses. ^1^ Mean ± SD or *n* (%). ^2^ Wilcoxon rank sum test or Fisher’s exact test. ^3^ One patient was excluded because pre-NST MRI was not available. pCR, pathological complete response; luminal, estrogen receptor (ER)-positive; HER2+, ER-negative and human epidermal growth factor receptor-2-positive; TN, triple-negative; NST, neoadjuvant systemic treatment; NME, non-mass enhancement; CR, complete response on MRI; CS, concentric shrinkage; and SD, stable disease on MRI.

**Table 3:** The diagnostic performances for the detected residual lesion across the two readers.

Population	Protocol	Reader	AUC	Sensitivity	Specificity	PPV	NPV
All	UF	1	0.86 (0.77–0.96)	0.91 (0.76–0.98)	0.82 (0.60–0.95)	0.88 (0.71–0.98)	0.86 (0.65–0.96)
		2	0.88 (0.79–0.97)	0.94 (0.80–0.99)	0.82 (0.60–0.95)	0.89 (0.72–0.99)	0.90 (0.70–0.97)
	Early	1	0.80 (0.69–0.90)	1.00 (0.89–NA)	0.59 (0.36–0.79)	0.79 (0.59–NA)	1.00 (0.77–1.00)
		2	0.68 (0.58–0.78)	1.00 (0.89–NA)	0.36 (0.17–0.59)	0.70 (0.46–NA)	1.00 (0.67–1.00)
	Delayed	1	0.70 (0.60–0.81)	1.00 (0.89–NA)	0.49 (0.21–0.64)	0.72 (0.49–NA)	1.00 (0.70–1.00)
		2	0.68 (0.58–0.78)	1.00 (0.89–NA)	0.36 (0.17–0.59)	0.70 (0.46–NA)	1.00 (0.67–1.00)
	HR	1	0.70 (0.60–0.81)	1.00 (0.89–NA)	0.41 (0.21–0.64)	0.72 (0.49–NA)	1.00 (0.70–1.00)
		2	0.68 (0.58–0.78)	1.00 (0.89–NA)	0.36 (0.17–0.59)	0.70 (0.46–NA)	1.00 (0.67–1.00)
Luminal	UF	1	0.86 (0.69–1.02)	0.96 (0.80–1.00)	0.75 (0.35–0.97)	0.92 (0.68–1.00)	0.86 (0.50–0.98)
		2	0.88 (0.71–1.03)	1.00 (0.86-NA)	0.75 (0.35–0.97)	0.93 (0.69–NA)	1.00 (0.60–NA)
	Early	1	0.88 (0.71–1.04)	1.00 (0.86-NA)	0.75 (0.35–0.97)	0.93 (0.61–NA)	1.00 (0.60–1.00)
		2	0.81 (0.63–0.99)	1.00 (0.86-NA)	0.63 (0.25–0.92)	0.89 (0.62–NA)	1.00 (0.56–1.00)
	Delayed	1	0.75 (0.56–0.94)	1.00 (0.86-NA)	0.50 (0.16–0.84)	0.86 (0.54–NA)	1.00 (0.50–1.00)
		2	0.81 (0.63–0.99)	1.00 (0.86-NA)	0.63 (0.25–0.92)	0.89 (0.62–NA)	1.00 (0.56–1.00)
	HR	1	0.75 (0.56–0.94)	1.00 (0.86–NA)	0.50 (0.16–0.84)	0.86 (0.54–NA)	1.00 (0.50–1.00)
		2	0.81 (0.63–0.99)	1.00 (0.86-NA)	0.63 (0.25–0.92)	0.89 (0.62–NA)	1.00 (0.56–1.00)
TN	UF	1	0.83 (0.64–1.02)	0.86 (0.42–1.00)	0.80 (0.44–0.98)	0.75 (0.37–0.99)	0.89 (0.49–0.99)
		2	0.76 (0.53–0.98)	0.71 (0.29–0.96)	0.80 (0.44–0.98)	0.71 (0.33–0.96)	0.80 (0.40–0.98)
	Early	1	0.80 (0.64–0.96)	1.00 (0.59–NA)	0.60 (0.26–0.88)	0.63 (0.29–NA)	1.00 (0.55–1.00)
		2	0.65 (0.50–0.80)	1.00 (0.59–NA)	0.30 (0.07–0.65)	0.50 (0.14–NA)	1.00 (0.38–1.00)
	Delayed	1	0.82 (0.54–0.86)	1.00 (0.59–NA)	0.40 (0.12–0.74)	0.54 (0.20–NA)	1.00 (0.45–1.00)
		2	0.65 (0.50–0.80)	1.00 (0.59–NA)	0.30 (0.07–0.65)	0.50 (0.14–NA)	1.00 (0.38–1.00)
	HR	1	0.82 (0.54–0.86)	1.00 (0.59–NA)	0.40 (0.12–0.74)	0.54 (0.20–NA)	1.00 (0.45–1.00)
		2	0.65 (0.50–0.80)	1.00 (0.59–NA)	0.30 (0.07–0.65)	0.50 (0.14–NA)	1.00 (0.38–1.00)
HER2+	UF	1	0.50	1.00 (0.03–NA)	0.00 (NA–0.60)	0.20 (NA)	NA (0.00–1.00)
		2	1.00	1.00 (0.03–NA)	1.00 (0.40–NA)	1.00 (0.14–NA)	1.00 (0.09–NA)
	Early	1	0.63	1.00 (0.03–NA)	0.25 (0.01–0.81)	0.25 (0.01–NA)	1.00 (0.03–1.00)
		2	0.63	1.00 (0.03–NA)	0.25 (0.01–0.81)	0.25 (0.01–NA)	1.00 (0.03–1.00)
	Delayed	1	0.63	1.00 (0.03–NA)	0.25 (0.01–0.81)	0.25 (0.01–NA)	1.00 (0.03–1.00)
		2	0.50	1.00 (0.03–NA)	0.00 (NA–0.60)	0.20 (NA)	NA (0.00–1.00)
	HR	1	0.63	1.00 (0.03–NA)	0.25 (0.01–0.81)	0.25 (0.01–NA)	1.00 (0.03–1.00)
		2	0.50	1.00 (0.03–NA)	0.00 (NA–0.60)	0.20 (NA)	NA (0.00–1.00)

The numbers in parentheses are the 95% confidence interval. Luminal, estrogen receptor (ER)-positive; HER2+, ER-negative and human epidermal growth factor receptor-2-positive; TN, triple-negative; UF, ultrafast dynamic contrast-enhanced MRI; HR, high-resolution contrast-enhanced MRI; AUC, the area under the receiver operating characteristic curve; PPV, the positive predictive value; NPV, the negative predictive value; and NA, not applicable.

## Data Availability

The data presented in this study are available on request from the corresponding author due to institutional ethical restrictions concerning patient privacy. Only anonymized, analyzed data (excluding patient identifiable information and images) can be considered for sharing, contingent upon review and approval by our institutional review board.
