# Correction: Honda et al. Visual Evaluation of Ultrafast MRI in the Assessment of Residual Breast Cancer After Neoadjuvant Systemic Therapy: A Preliminary Study Association with Subtype. *Tomography* 2022, *8*, 1522–1533

**DOI:** 10.3390/tomography12010006

**Published:** 2026-01-06

**Authors:** Maya Honda, Masako Kataoka, Mami Iima, Rie Ota, Akane Ohashi, Ayami Ohno Kishimoto, Kanae Kawai Miyake, Marcel Dominik Nickel, Yosuke Yamada, Masakazu Toi, Yuji Nakamoto

**Affiliations:** 1 Department of Diagnostic Imaging and Nuclear Medicine, Kyoto University Graduate School of Medicine, 54 Shogoin-Kawaharacho, Kyoto 606-8507, Japan; mayah.217@gmail.com (M.H.); mamiiima@kuhp.kyoto-u.ac.jp (M.I.); ota_rie0624@kuhp.kyoto-u.ac.jp (R.O.); kanaek@kuhp.kyoto-u.ac.jp (K.K.M.); ynakamo1@kuhp.kyoto-u.ac.jp (Y.N.); 2 Department of Diagnostic Radiology, Kansai Electric Power Hospital, 2-1-7, Fukushima, Osaka 553-0003, Japan; 3 Institute for Advancement of Clinical and Translational Science (iACT), Kyoto University Hospital, 4 Shogoin-Kawaharacho, Kyoto 606-8507, Japan; 4 Department of Translational Medicine, Diagnostic Radiology, Lund University, Skåne University Hospital, 205-02 Malmo, Sweden; amaoh135@gmail.com; 5 Department of Diagnostic Radiology, Kyoto Katsura Hospital, Yamadahirao-cho, Kyoto 615-8256, Japan; akohno1980@gmail.com; 6 MR Application Predevelopment, Siemens Healthcare GmbH, Allee am Roethelheimpark 2, 91052 Erlangen, Germany; marcel.nickel@siemens-healthineers.com; 7 Department of Diagnostic Pathology, Kyoto University Hospital, 54 Shogoin-Kawaharacho, Kyoto 606-8507, Japan; yyamada@kuhp.kyoto-u.ac.jp; 8 Department of Breast Surgery, Kyoto University Graduate School of Medicine, 54 Shogoin-Kawaharacho, Kyoto 606-8507, Japan; toi@kuhp.kyoto-u.ac.jp

This correction addresses several errors identified in the original publication [1].

1. Definitions of Abbreviations

The definitions for previously undefined abbreviations have been added to the manuscript as follows:

- In the Abstract, Section 2.4 and Results, “ROC” and “AUC” were expanded to “receiver operating characteristic (ROC)” and “area under the ROC curve (AUC)”, respectively.
- In the Abstract, Introduction and Results, “DCE” was expanded to “dynamic contrast-enhanced (DCE)”.
- In the Introduction, “CR” and “HER2” were expanded to “complete response (CR)” and “human epidermal growth factor receptor 2 (HER2)”, respectively.
- In Section 3, “CDK 4/6” was expanded to “cyclin-dependent kinases 4 and 6 (CDK 4/6)”.
- In the Figure 1 legend, “UF-DCE” was expanded to “ultrafast DCE”.
- In the Figure 2 legend, the text “UF-DCE MRI: ultrafast DCE MRI” has been corrected to “UF-DCE MRI: ultrafast dynamic contrast-enhanced MRI”.
- In the Figure 7 legend, “CR” and “ultrafast-DCE” have been corrected to “complete response” and “ultrafast dynamic contrast-enhanced”, respectively.
- In the Figure 8 legend, “CDK 4/6” and “DCE” have been corrected to “cyclin-dependent kinases 4 and 6” and “dynamic contrast-enhanced”, respectively.

2. Correction of Numerical Data in Results Section

The previously published number of lesions achieving pathological complete response (pCR) was incorrect.

A correction has been made to the first paragraph in Results section:

A total of 55 lesions in 55 patients were evaluated, of which 22 lesions achieved pCR.

3. Corrections of Table 2

Table 2 as published contained a mistake. The data columns for pCR and non-pCR was reversed. The corrected Table 2 appears below.

4. Data Availability Statement

The incorrect Data Availability Statement (“not applicable”) was revised to accurately reflect the availability of our research data. The updated statement is as follows: The data presented in this study are available on request from the corresponding author due to institutional ethical restrictions concerning patient privacy. Only anonymized, analyzed data (excluding patient identifiable information and images) can be considered for sharing, contingent upon review and approval by our institutional review board.

The authors state that the scientific conclusions are unaffected. These corrections were approved by the Academic Editor. The original publication has also been updated.

## Figures and Tables

**Table 2:** The patient characteristics.

Variables	*n*	Overall, *n* = 55 ^1^	pCR, *n* = 22 ^1^	Non-pCR, *n* = 33 ^1^	*p*-Value ^2^
Age (years)	55	49.7 ± 11.7	48.6 ± 9.7	50.5 ± 12.9	0.8
Subtype	55				0.007
Luminal		33 (60%)	8 (36%)	25 (76%)	
HER2+		5 (9.1%)	4 (18%)	1 (3.0%)	
TN		17 (31%)	10 (45%)	7 (21%)	
pre-NST size (mm)	54 ^3^	40.5 ± 23.7	28.9 ± 19.8	48.5 ± 23.0	<0.001
Morphology	54 ^3^				0.5
mass		43 (80%)	19 (86%)	24 (75%)	
NME		11 (20%)	3 (14%)	8 (25%)	
Shrink pattern	54 ^3^				0.004
CR		5 (9.3%)	5 (23%)	0 (0%)	
CS		24 (44%)	12 (55%)	12 (38%)	
non-CS		17 (31%)	4 (18%)	13 (41%)	
SD		8 (15%)	1 (4.5%)	7 (22%)	

Data in columns 2 and 3 are the number of patients with the percentage in parentheses. ^1^ Mean ± SD or *n* (%). ^2^ Wilcoxon rank sum test or Fisher’s exact test. ^3^ One patient was excluded because pre-NST MRI was not available. pCR, pathological complete response; luminal, estrogen receptor (ER)-positive; HER2+, ER-negative and human epidermal growth factor receptor-2-positive; TN, triple-negative; NST, neoadjuvant systemic treatment; NME, non-mass enhancement; CR, complete response on MRI; CS, concentric shrinkage; and SD, stable disease on MRI.
